# Effects of Graphite on Electrically Conductive Cementitious Composite Properties: A Review

**DOI:** 10.3390/ma14174798

**Published:** 2021-08-24

**Authors:** Ting Luo, Qiang Wang

**Affiliations:** Department of Civil Engineering, Tsinghua University, Beijing 100084, China; luot20@mails.tsinghua.edu.cn

**Keywords:** ECCCs, graphite, dispersion, workability, durability, conductive mechanism, electrical conductivity

## Abstract

Electrically conductive cementitious composites (ECCCs) have been widely used to complete functional and smart construction projects. Graphite, due to its low cost and wide availability, is a promising electrically conductive filler to generate electrically conductive networks in cement matrixes. Cement-based materials provide an ideal balance of safety, environmental protection, strength, durability, and economy. Today, graphite is commonly applied in traditional cementitious materials. This paper reviews previous studies regarding the effects and correlations of the use of graphite-based materials as conductive fillers on the properties of traditional cementitious materials. The dispersion, workability, cement hydration, mechanical strength, durability, and electrically conductive mechanisms of cementitious composites modified with graphite are summarized. Graphite composite modification methods and testing methods for the electrical conductivity of ECCCs are also summarized.

## 1. Introduction

Cement is a dielectric material which functions as an ionic conductor due to its water content [1]. Electronic conduction can also be engineered by adding electrically conductive fillers to cement-based materials [2]. Various types of conductive fillers can reduce the electrical resistivity of cement-based materials so as to realize electrical conductivity. Carbon-based fillers have been widely investigated in recent decades. Cementitious composite materials with high electrical conductivity can be obtained by modification with conductive carbon fillers such as graphite power (GP) [3], graphene [4], carbon nanotubes (CNTs) [5], carbon fibers (CFs) [6], and carbon black (CB) [7] to form conductive networks inside the cementitious matrix [8].

**Figure 1:**
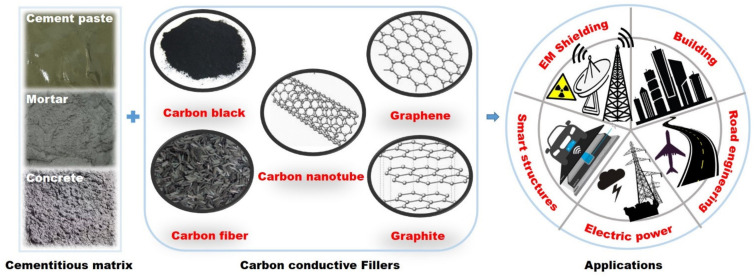
Potential applications of conductive carbon material within ECCCs.

The addition of functional fillers can also endow some properties of electrically conductive cementitious composites (ECCCs), such as the electromagnetic (EM) shielding effect [8,9,10,11]. The multifunctionality of ECCCs lends wide application prospects in terms of de-icing and snow melting [12,13,14], EM shielding of vital equipment [15], electric grounding materials [16], cathodic protection systems [17], structure health monitoring systems [18,19], and self-sensing for smart structures [20,21]. Figure 1 shows a diagram of potential applications of conductive carbon material within ECCCs.

The electrical conductivity of cementitious composites is controlled by the conductivity of the conductive filler itself, the dispersion degree of the filler components, and the contact resistivity of the interface between the filler phase and the matrix [22]. Graphite has a stacked planar sp^2^-hybridized C_6_ ring structure [23] with excellent electrical, thermal, and mechanical properties [24,25,26]; it has proven to be an excellent conductor of electricity [27]. Compared with other carbon allotropic forms (2D graphene, 1D CNTs, 1D CFs, and 0D CB), 3D GP is an ideal electric conduction phase for improving the electrical and mechanical properties of cement-based materials [3,23,28].

CB is less crystalline than GP, so it is less conductive. Further, graphene, CNTs, and CFs are more expensive than GP. Fiber fillers do not disperse as readily as powder fillers as the high aspect ratio of fibers gives them the tendency to cling together [22]. Dispersion has important effects on both the electrical and mechanical properties of composites. An overview of the general properties of carbon materials is given in Table 1. The practical application of high-performance carbon materials is restricted by their high cost, so this article mainly centers on the research progress of graphite, which is relatively inexpensive.

Graphite is widely considered to be a prospective material in certain cases [29] and a critical material in other cases for both industrial and national security applications [23]. Graphite has been tested as a conductive filler to find that it can improve electrical conductivity performance. Ioanna et al. [3] reported that GP has a layered planar structure, rendering it relatively soft due to its anisotropy and weak inter-planar forces; it also conducts electricity and heat well, is resistant to chemical attacks, and remains stable under standard conditions. Chen et al. [30] reported that graphite can fill the space between fibers and form a local conductive network. The synergistic effect of electron conduction and electron transition increases the intelligent agility of conductive concrete. Bhattacharya et al. [31] reported a novel conductor–insulator composite system designed with graphite-filled cement composites. The system showed high mechanical strength and strong shielding effectiveness against electromagnetic radiation.

**Figure 2:**
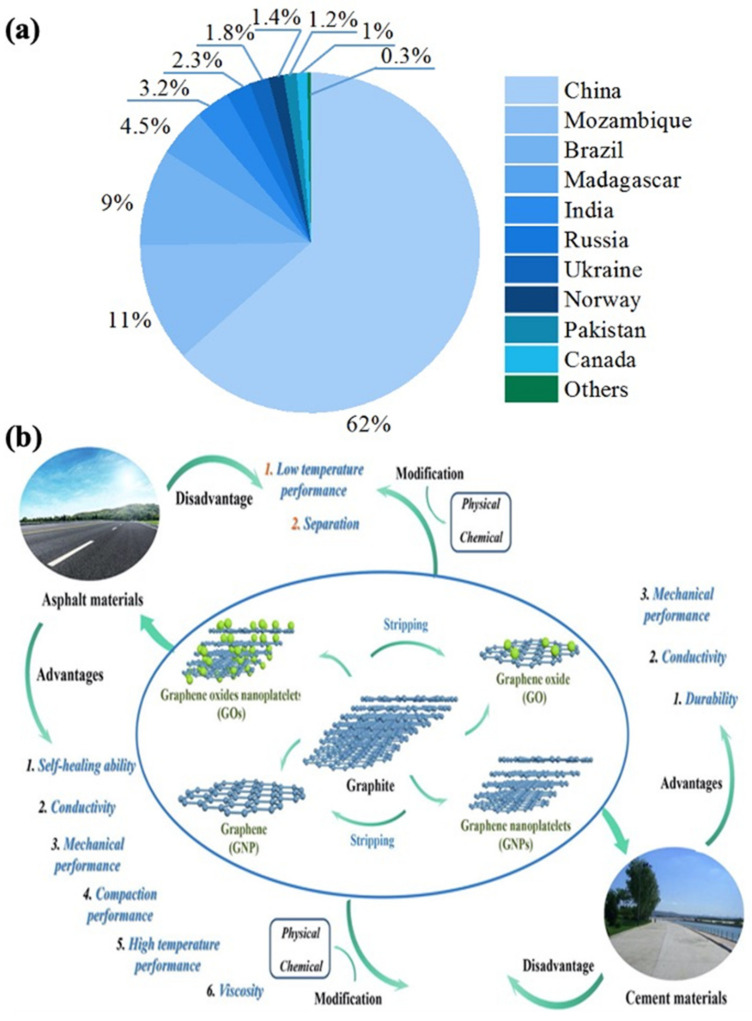
(**a**) Countries as major producers of graphite; (**b**) main applications of graphite-based materials in civil engineering (reprinted from [32] ©2021 with permission from Elsevier).

Based on data from the US Geological Survey (Mineral Commodity Summaries—2021), the total global production of graphite was about 1.05 million tons in 2020. Major producers of graphite and the primary applications of graphite-based materials in civil engineering are shown in Figure 2a,b, respectively. Graphite is a national strategic supply material representative of a 21st-century sunrise industry, with very extensive application fields. The emergence of electric vehicles and a continuous increase in demand for green energy have dramatically revolutionized the graphitic carbon market [23]. Demand for graphite is expected to continue growing rapidly. Various types of graphite-based materials lend different properties to different types of cement and induce varied, unique properties into cementitious composite materials [3,4,5,10,16,27,28,30]. Hence, graphite-based materials are potentially applicable in large-scale civil engineering projects.

**Figure 3:**
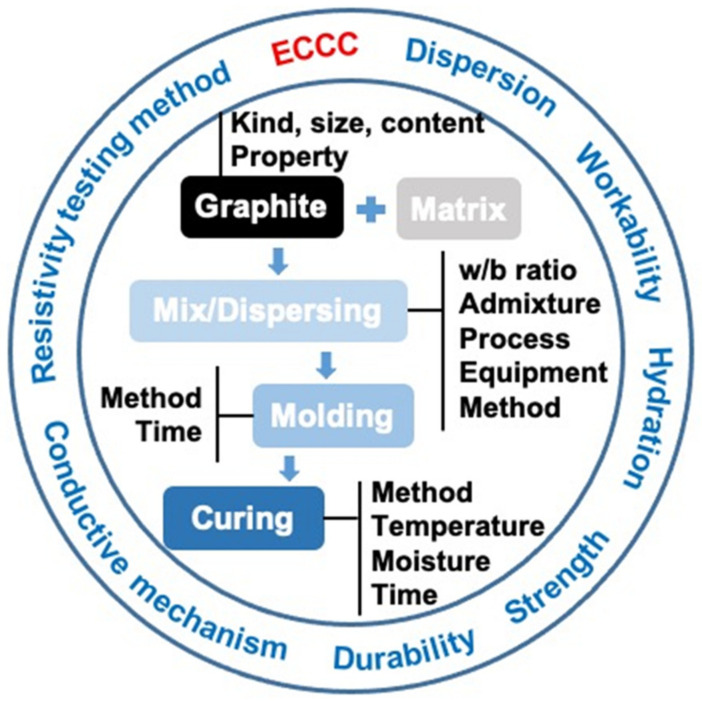
Schematic representation of main topics of this review.

To date, although several important reviews have discussed ECCCs [8,22], most have focused on multi-element conductive fillers rather than specifically targeting graphite-based ECCCs. Our focus here is on the properties of the cementitious composites mixed with graphite in fresh and hardened states. As shown in Figure 3, this review covers the dispersion, workability, cement hydration, mechanical strength, durability, and electrical conductivity of traditional cementitious materials modified with graphite. Various modify graphite composite modification methods and testing methods for the electrical conductivity of ECCCs are also summarized.

## 2. Inherent Properties of Graphite

**Figure 4:**
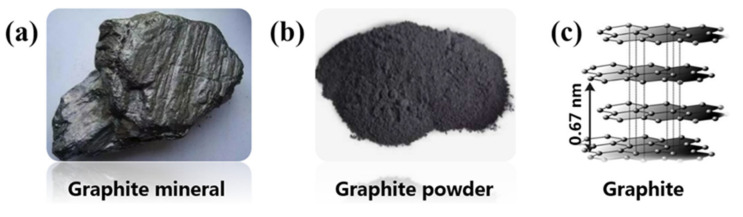
Photographs of (**a**) graphite mineral, (**b**) flake graphite powder, (**c**) crystalline structure of graphite.

Graphite can be divided into two categories: natural and artificial. Natural graphite is a mineral as shown in Figure 4a, which is found in metamorphic rocks and igneous rocks with extremely soft sheets and very low specific gravity [33]. Artificial graphite affords various properties of the material due to the different types of precursors and formation processes [23]. The most common form of graphite currently utilized is flake graphite, which is suitable for many practical applications and has the highest market share in the world among the various forms of graphite available [34]. Photographs of the crystalline structure of flake graphite powder are shown in Figure 4b,c. The fundamental structure of graphite is composed of a series of stacked parallel layers (i.e., graphene layers), which are comprised of carbon atoms bonded by strong covalent bonds. Weak bonds (Van der Waals) also exist among each layer. The d-spacing of the C_6_ ring is 0.335 nm [35,36,37].

The fundamental structure of graphite determines its anisotropy. In-plane metallic bonding provides strong electrical and thermal conductivity within its layers, while weak Van der Waals forces among the layers result in poor electrical and thermal conductivity perpendicular to them [38,39]. The anisotropy allows the carbon layers to slide easily over each other, thus making graphite a highly lubricating material [38]. High chemical inertness, corrosion resistance, large heat capacity, and high thermal structural stability ensure diverse technological applications among graphite-based materials [40,41,42]. Graphite is also a natural conductive filler, making it a popular material for preparing conductive composites [43]. However, the applications of graphite-based ECCCs are limited, mainly because conductive fillers cause poor workability and deteriorate mechanical strength and durability.

The successful use of graphite in ECCCs requires: (1) adequate dispersion in the aqueous fresh mix to ensure that an electrically conductive network forms within the cementitious structure [44]; (2) sufficient workability to ensure wide application in practical engineering projects [45]; and (3) adequate bonding of cement hydration products to surfaces for effective stress transfer across the interfaces, thus securing proper ECCC mechanical properties [46].

## 3. Dispersion of Graphite in Cement-Based Materials

**Figure 5:**
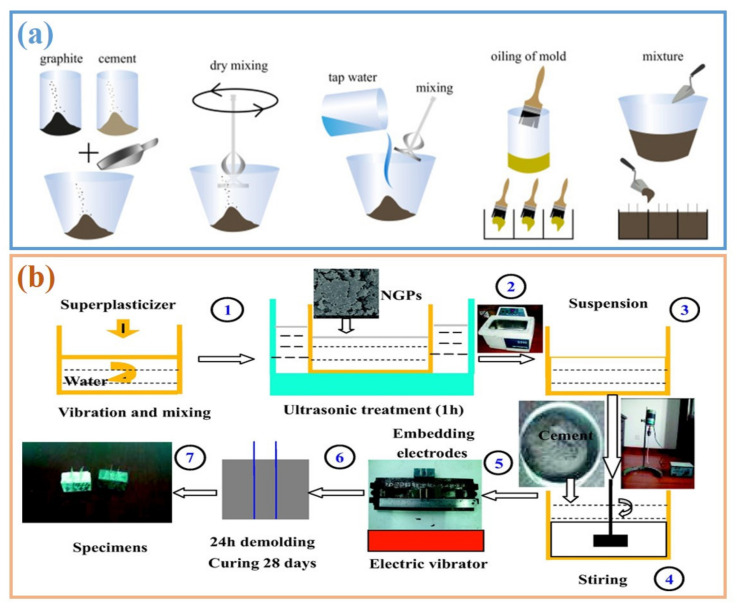
Preparation of graphite filled cementitious composites. Dispersion methods of (**a**) mixing compounds in dry powder form (reprinted (adapted) from [50] which is an open access article and permits unrestricted use); (**b**) adding powders into solution to prepare uniform suspensions (reprinted (adapted) with permission from [51] ©2017 with permission from Elsevier).

The dispersion of graphite is a problem in regard to the properties of graphite composites [18,47]. The surfaces of graphite are hydrophobic and atomically smooth, which lead to mutual bonding (i.e., agglomeration) in aqueous solutions (e.g., fresh cement mixtures) [48]. The agglomeration of graphite within a cementitious system prevents it from fully forming conductive networks. It is not feasible to disperse graphite directly within cement paste during the mixing process, as paste thickens very quickly upon the addition of water [49]. In general, there are two strategies for adding the graphite powders into a cement matrix: dry mixing dispersal in the solid phase, or ultrasonic dispersal in the liquid phase. The preparation process of cementitious composites with graphite fillers is illustrated in Figure 5a,b.

The dispersion of particles is largely determined by their free surface energy as well as the polar and dispersive parts of their components [52]. Therefore, non-polar carbon-based materials such as graphite do not readily disperse in highly polar media such as water [53]. The poor or insufficient dispersion of fillers results in large clusters within the hydrated paste that negatively affect the properties of the cement matrix [44,52,53,54]. Various approaches have been employed to improve the dispersibility of carbon material in cementitious matrices, such as the use of surfactants [55], cement admixtures [53,56], and surface modifications [57,58].

**Figure 6:**
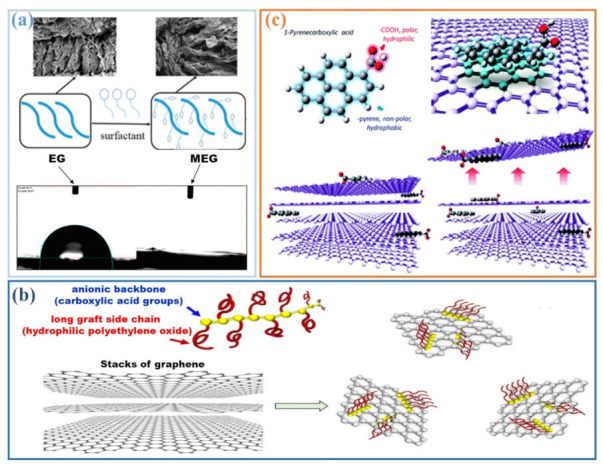
Homogeneous dispersion of graphite via (**a**) surfactants (reprinted (adapted) with permission from [59]. Copyright © 2017 American Chemical Society); (**b**) cement admixtures (reprinted (adapted) with permission from [61] © 2018 with permission from Elsevier); (**c**) surface modifications (reprinted (adapted) with permission from [62]. Copyright © 2010 American Chemical Society).

Commonly used surfactants include sodium dodecylbenzene sulfonate (SDBS), cetyl trimethyl ammonium bromide (CTAB), sodium deoxycholate (NaDC), gum Arabic (GA), and Triton X-100 (TX100). The aqueous dispersion of hydrophobic materials can be improved with surfactants by reducing the surface tension of water. Zhou et al. [59] employed TX100 to modify expanded graphite (EG) for improved hydrophilicity. As shown in Figure 6a, the contact angle of EG with water is about 87.1° while that of TX100 modified EG (MEG) is around 0°. To this effect, modification with TX100 is effective for EG.

Commonly used cement admixtures include polycarboxylate superplasticizer (SP), naphthalene superplasticizer (NS), and lignosulfonate (L), which are used as water-reducing agents within cement paste. Wang et al. [60] investigated the dispersion of graphene nanoplatelets (GNPs) using different water reducing agents with a sulfonic group (-SO_3_H), hydroxyl (-OH), and amino group (-NH_2_), respectively. The results showed that these groups can be grafted onto the surface of GNPs, weakening the interaction between the graphene layers to further improve their hydrophilicity and dispersion. As shown in Figure 6b, Du et al. [61] found that SP molecules can absorb onto the surface of GNPs as the polarity of the GNP itself is similar to the anionic backbone in the SP molecule. The hydrophilicity of the long, grafted side chains of the SP molecule can effectively prevent GNP from agglomerating in water, thus enhancing dispersion. As a result, GNP sheet layers can be gradually separated from the GNP agglomerates. 

Surface modification techniques based on the introduction of hydrophilic groups to graphite can improve its dispersion in aqueous media. An et al. [62] used 1-pyrenecarboxylic acid (PCA) to modify the surface of graphene sheets to provide a polar medium for stable dispersion. As shown in Figure 6c, PCA has a hydrophobic (nonpolar) pyrene group and a hydrophilic (polar) carboxylic acid group (-COOH). Hence, PCA can interact with an exposed graphitic surface through the hydrophobic pyrene group while the hydrophilic -COOH enables the sheets to be dispersed in water as a complex.

The fabrication of graphite-cement (GC) composites is the key to the feasibility and applicability of ECCCs. However, chemical pathways cannot directly disperse graphite materials in water. Rather, they can be targeted to improve dispersion and stability by wetting the graphite materials with water. Typically, these chemical pathways used in combination with ultrasonic treatment to directly disperse the graphite material.

## 4. Workability of Graphite in Cement-Based Materials

Conductive cementitious composite is a heterogeneous material. Its poor workability restricts its practical application [44]. “Workability” refers here to the ease of flow and consolidation in fresh cement composites, which significantly affects the mechanical properties and durability of hardened cement composites. Many researchers have reported that the addition of graphite adversely affects workability (Table 2).

Increasing the content [18] or fineness [3] of graphite drastically reduces its fluidity due to the inter-particle friction with cement particles and the low hydrophilicity of graphite [3,63]. Wang et al. [44] reported that the spread diameter of cement paste with 4% graphite content is reduced by about 50% and the shear stress is increased by 300%. The poor fluidity of GC paste can be attributed to water trapped in agglomerated graphite particles, which decreases the amount of free water. El-Dieb et al. [18] reported that the incorporation of 7% graphite (by volume) can reduce slump by 33%; the slump reduction was in a nearly linear relationship with the increase in graphite replacement level due to the very high surface area of graphite. Domenico et al. [64] found that when graphite content increases to a certain extent (>70%), GC composites have no consistency and quickly collapse into powder.

Ioanna et al. [3] investigated the effect of graphite fineness on cementitious composite performance. The viscosity of the samples with 10% (by weight) coarse graphite and fine graphite increased progressively by 76% and 130% compared to their control, respectively. Fine graphite of the same weight dosage has more particles that cause inter-particle friction with cement, which dramatically increases viscosity. Moreover, smaller size graphite has a relatively large surface area that requires more water to cover. Wang et al. [60] used different water-reducing agents to disperse GNPs, improve their fluidity, and promote electrostatic repulsion and steric repulsion among particles. They successfully reduced the adsorption of water to partially mitigate the negative impact of GNPs on fluidity [65].

Overall, a reduction in fluidity creates practical limitations when using graphite as a conductive additive. The poor workability is a significant barrier that restricts its wide application in practical engineering. The workability of fresh ECCC is of key importance to ensure the quality and mechanical performance of the harden ECCC. Hence, the mixture design, water content, water reducing agent utilized, graphite content, and fineness must be adjusted to ensure the sufficient flowability without affecting functionality. 

**Table 2:** Influence of graphite on fresh cement composite workability.

Matrix	Graphite Content	w/c	Method	Changes in Fluidity/Slump	Refs.
Paste	10, 20, 30 and 40 (wt%) ^a^	0.45	Rheology measurement	Increasing graphite fineness led to a dramatic reduction in fluidity. Viscosity increased progressively as graphite content increased.	[3]
Concrete	0.23, 0.68, 1.13, and 1.58 (vol%) ^b^	0.57	Slump tests	The effect of graphite on slump increased as the replacement level increased. The use of 7 vol% replacement resulted in 33% reduction in slump.	[18]
Paste	1, 2, 3 and 4 (wt%) ^a^	0.5	Mini-cone test	Spread diameters decreased with increase in graphite addition.	[44]
Mortar	0.01, 0.1, and 0.2 (wt%) ^a^	0.18	Rheology measurement	Nano-graphite thickened the cementitious admixture.	[66]
Paste	10, 15,20, and 30 (wt%) ^a^	0.4	Flow diameter test	Cement paste flow diameter decreased from 25.5 cm to 9.5 cm after 30% graphite addition.	[67]
Mortar	10, 20, 30 (wt%) ^a^	0.7	Flow diameter test	The flow diameter of 25.4 cm for plain cement mortar reduced to 12.5 cm when graphite was increased to a 30% weight.	[67]
Mortar	Graphite nanoplatelets water paste	-	Rheology measurement	A higher shear stress to start flowing and a slightly higher plastic viscosity were observed. Workability decreased due to the reduction of free water in the paste and an increase in friction among the particles.	[68]

^a^: By weight of cement; ^b^: by volume of total concrete.

## 5. Effect of Graphite on Cement Hydration

Hydration kinetics play an important role in the microstructural development and final properties of cement composites [69]. The hydration products of cement generally include hydrated calcium silicate gels (C-S-H), calcium hydroxide (CH), ettringite (AFt), and monosulfates (AFm). Many researchers [3,70,71] have reported that graphite does not directly participate in hydration; cement hydration is not affected by graphite addition when using it as a conductive additive.

**Figure 7:**
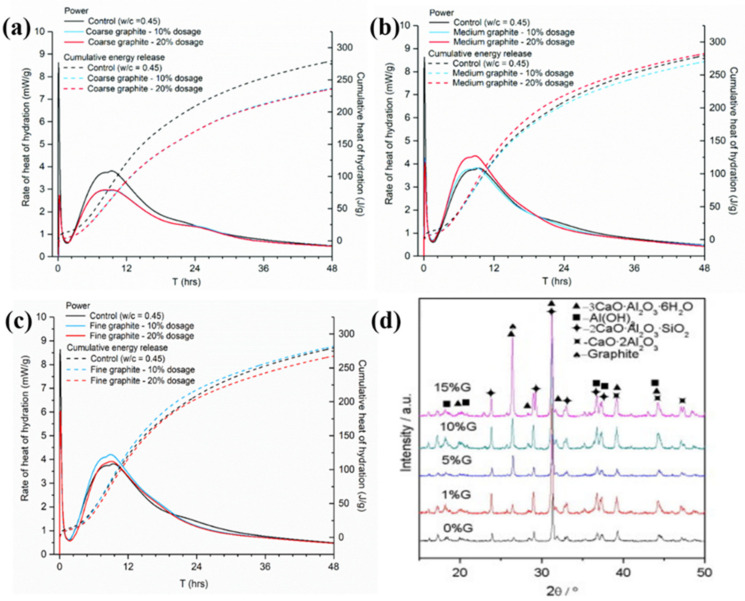
Effect of graphite size and concentration on cement paste hydration: (**a**) coarse, (**b**) medium, and (**c**) fine graphite (reprinted (adapted) from [3] which is an open access article and permits unrestricted use); (**d**) XRD patterns of graphite-aluminate cement composite paste (reprinted (adapted) with permission from [71] ©2012 with permission from Elsevier).

Tadahiro et al. [70] quantitatively analyzed hardened GC paste hydrates containing graphite to find that the amount of CH was in proportion to the initial amount of cement, and that the Ca/Si molar ratio in C-S-H was constant. To this effect, graphite appears to not directly participate in hydration. Ioanna et al. [3] reported that graphite in a cement matrix acts as an inert filler. With increasing graphite fineness (>100 mesh), the filler effect emerges, and fine graphite begins to promote cement hydration due to the hydrophobic graphite particles pushing water towards the cement grains. The effects of graphite on the hydration process can be observed by isothermal calorimetry measurement, as shown in Figure 7a–c. As illustrated in Figure 7a–c, the same hydration peaks were observed in all cases, which indicated that graphite acts as an inert filler and does not participate directly in cement hydration. However, the three graphite products had a somewhat different effect on the hydration. The differences between fineness products can be explained by their physical mechanisms. The evolution of hydration products of aluminate cement mixed with graphite was analyzed by Yuan et al. [71] The XRD results shown in Figure 7d indicate that the characteristic peak of graphite is enhanced as graphite content increases, while the corresponding peak positions and intensities of other phases remain constant. To this effect, graphite does not directly participate in the hydration process.

**Figure 8:**
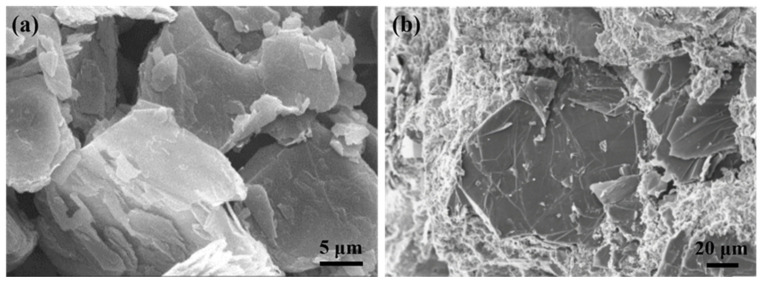
SEM micrographs of (**a**) pure graphite (reprinted (adapted) with permission from [71] ©2012 with permission from Elsevier) and (**b**) graphite-cement paste after five months (reprinted (adapted) from [3] which is an open access article and permits unrestricted use).

Zel et al. [72] used the neutron diffraction method to analyze the primary phase of graphite-cement composite materials. The results diffraction peaks corresponding to graphite, AFt, and CH phases, respectively, with no extra new crystal phase produced. Bi et al. [73] reported that nucleation sites can be provided for hydration product precipitation due to the addition of carriers with a large surface area. Graphite sheets have large and thin flake structures (Figure 8a) which can act as nucleation sites in GC composites to promote the nucleation and growth of hydration products. Figure 8b shows the microstructural characterization of five-month-old GC paste by SEM. A large number of hydration products (mainly C-S-H and a small amount of Aft) can be observed near the graphite flakes, which may be attributable to the high surface area of graphite [3]. The addition of graphite does not significantly affect the cement matrix, which indicates close compatibility of graphite with cement composites [67].

## 6. Effects of Graphite on Cementitious Composite Mechanical Performance

The mechanical properties of cement composites are a critical indicator of performance in many applications [69]. Conductive fillers (e.g., graphite) may be added in efforts to provide satisfactory conductivity in concrete but can drive down the strength of the material [45]. Many studies have shown that graphite influences the mechanical properties of GC composites (Table 3). Compressive strength decreases after graphite addition at all test ages, and to a greater extent as the graphite dosage increases.

El-Dieb et al. [18] reported that concrete with strong conductivity can be produced by adding appropriate types and contents of conductive fillers as partial replacements for the fine aggregate, but their use negatively impacts compressive strength. Wu et al. [74] reported that graphite does not improve the strength of conductive composites due to its intrinsic structural features (Figure 4c). Ioanna et al. [3] used micro-indentation testing to assess the effects of graphite on the mechanical performance of cement paste; they found that hardness decreases after graphite addition. Frattini et al. [64] reported that when graphite addition exceeds 40%, the compressive strength of GC composites is less than 5 MPa.

Previously reported strength reduction mechanisms for graphite include the following. (1) Cementitious matrix and graphite particles have poor adhesion, so the porosity of hardened GC composites tends to increase [18,46,70]. (2) Loose bonding among graphite sheets allows the graphite to easily slip between layers, which damages the microstructures and drives down mechanical properties [74]. (3) Water is entrapped in agglomerated graphite particles and blocked from reaching the cement grains [3,44]. (4) The use of graphite increases the demand for water, which reduces concrete strength [45]. (5) The agglomeration of graphite is not conducive to the mechanical properties of cement paste [44]. (6) Strength and density are reduced when graphite replaces cement or sand [67]. 

The conductive network formed by graphite sheets plays an important role in the electrical conductivity of GC composites, but also degrades mechanical properties. The graphite content should be controlled within an appropriate level to prepare a matrix with both electrical conductivity and strong mechanical performance [74].

**Table 3:** Effects of graphite on cement-based material mechanical performance.

Matrix	w/c	Graphite Size (μm)	Compressive Strength		Ref.
			Graphite Content	Increase/Reduction (%)/d	
Paste	0.45	2000	10 (wt%) ^a^	−47/2d, −39/7d, and −45/28d	[3]
		150		−28/2d, −32/7d, and −36/28d	
		44		−12/2d, −21/7d, and −12/28d	
		2000	20 (wt%) ^a^	−51/2d, −50/7d, and −56/28d	
		150		−31/2d, −40/7d, and −42/28d	
		44		−21/2d, −20/7d, and −35/28d	
Concrete	0.57	Few microns	0.23 (vol%) ^b^	−6/28d	[18]
			0.68 (vol%)	−17/28d	
			1.13 (vol%)	−22/28d	
			1.58 (vol%)	−30/28d	
Concrete	0.38	30	2.5 (wt%) ^a^	+10.1/28d	[30]
			5 (wt%)	+7.8/28d	
			7.5 (wt%)	+0.5/28d	
Paste	0.53	12 (d90)	5 (wt%) ^a^	−28/21d	[64]
	0.55		10 (wt%)	−39/21d	
	0.60		20 (wt%)	−61/21d	
	0.65		30 (wt%)	−72/21d	
	0.70		40 (wt%)	−83/21d	
	0.75		50 (wt%)	−93/21d	
	0.80		60 (wt%)	−95/21d	
	0.85		70 (wt%)	−97/21d	
	0.90		80 (wt%)	−98/21d	
Concrete	0.30	11 (d50)	5 (wt%) ^a^	−12/7d	[75]
			10 (wt%)	−38/7d	
			15 (wt%)	−49/7d	
			20 (wt%)	−52/7d	
Mortar	0.4	30	0.5 (wt%) ^a^	+1.2/28d	[76]
			1.0 (wt%)	−5.5/28d	
			2.0 (wt%)	−10.1/28d	
			3.0 (wt%)	−18.9/28d	
Concrete	0.59 0.801.0 1.2	1–5000	5.0 (wt%) ^c^ 10 (wt%) 15 (wt%) 20 (wt%)	−82.5/28d −91.9/28d −96.2/28d −99.4/28d	[77]

^a^: By weight of cement; ^b^: by volume of total concrete; ^c^: by weight of sand.

## 7. Effects of Graphite on Cementitious Composite Durability

The durability of cement-based cementitious materials refers to the resistance to environmental media (such as CO_2_, SO2-4, and Cl^-^) and the ability to maintain the desired properties and integrity long-term [78]. The durability of cement-based materials is directly related to their transport performance. The main penetration channels of erosive agents are cracks and pores within the cement matrix [69]. However, there have been relatively few studies to date on the durability of GC composites. This section summarizes the effects of graphite on the durability of cementitious materials as reported in the literature.

“Transport performance” is defined as the penetration rate of erosive agents (such as H_2_O and ions) into the cement matrix within the service environment [79]. Connected pores are inherent microstructural defects in cementitious materials that act as primary transmission channels. An open porosity test of GC composites was conducted by Medina et al. [67] to find that after adding 30% graphite, the open porosity of cement paste is 49.5% higher than that of plain cement. 

The carbonation of cementitious composites is a chemical corrosion process that reduces alkalinity in the cement matrix and causes corrosive damage to the material. A carbonation test of GC composites was also performed by Medina et al. [67] to find that the carbonation depth increases significantly with the addition of graphite. The ability of graphite to capture CO_2_ molecules and an increase in porosity in GC composites appeared to accelerate the movement of CO_2_ into the matrix.

Interestingly, other researchers have reported that water absorption is reduced after the immersion of graphite-cement composites. Peyvandi et al. [10] conducted acid resistance and moisture sorptivity tests to find that GNP incorporation enhances the moisture sorption resistance of concrete specimens. Medina et al. [67] reached a similar conclusion whereby the addition of graphite in their cement paste samples significantly reduced capillary absorption. Graphite likely reduces the accessibility to liquids and diminishes the size and tortuosity of the pore network. Previous studies [58,66,80] have reported that graphene-based materials in cementitious matrixes act as a physical barrier. The incorporation of graphite leads to the formation of tortuous network paths which ultimately decrease permeability. 

Carbon-based additives significantly improve the crack resistance of cementitious matrixes during exothermic reactions, especially in the initial stages of the hydration process [81,82]. The high surface area of the fillers allows them to efficiently control the propagation of microcracks in cementitious composite materials. Highly dense graphite powder is not prone to disintegration even under harsh experimental conditions (such as ion bombardment) [23], so its incorporation into cement-based materials may prevent calcium leaching under aggressive solutions (pH < 12.5). Mehdi et al. [66] found that the penetration of chloride decreases significantly as the amount of nano-graphite addition increases. Chloride ions may be entrapped in between the graphene layers of graphite [83], so an appropriate graphite addition can protect the matrix.

Previously published experimental results have highlighted the effects of graphene-based materials (graphite powder, nano-graphite, and graphite nanoplatelet) in regard to cementitious material durability based on graphite-containing composites. However, the long-term performance of graphite-based cementitious composites has not yet been reported (e.g., freeze–thaw resistance, shrinkage, sulfate resistance, steel corrosion resistance). Further studies on other durability related properties are needed to support the use of graphite in construction practice.

## 8. Electrical Properties of Graphite-Based ECCCs

Regular concrete is a poor conductor. The resistivity of saturated and dry concrete ranges between 10^6^ Ω cm and 10^9^ Ω cm, respectively [18,84]. It is theoretically feasible to obtain certain electrical properties in cementitious composite matrixes by adding different conductive materials [1,2,3,5,6,7,8,11,12,18,22,43,47,85]. Our focus in this section is the effects of graphite-based materials as conductive fillers. Electrical conductivity is the primary ECCC index that determines its performance and application value [86]. Studies have shown that changes in graphite content cause the resistivity of concrete to range from 10^−1^ Ω cm to 10^5^ Ω cm [45,64]. Graphite has considerable conductive capacity with its high carbon content (>98%), which can significantly enhance the conductivity of ECCCs [87].

Achieving high electrical conductivity in cementitious composites requires that conductive fillers be percolated through the cementitious matrix. Percolation is a common phenomenon in particle-filled composites, where certain physical properties (e.g., conductivity) of the system change suddenly when the concentration of the particles reaches a certain level [88]. This critical value is the “percolation threshold” to which the dosage of conductive fillers should be equal to or greater than in order to form conductive networks through the composites.

### 8.1. Conductive Mechanisms 

The conductive mechanisms of cementitious materials theoretically include conductive pathways, tunnelling effect, and field emission [8,89]. Current is transmitted in the cementitious matrix through electrons or holes in the conductive network and through tunnels over the substrate barrier after conductive fillers are added [45,88].

(1) Conductive pathway theory: when some conductive fillers are in contact with each other, the conductive pathway can be formed to allow current to pass through the cementitious matrix [90].
(2) Tunnelling effect theory: in a cementitious matrix, partially conductive fillers are distributed in the form of isolated particles or small aggregates. When these isolated particles and small aggregates are surrounded by a thin layer of hydration products, the electrons can hop across the thin layer into adjacent conductive particles [91]. This phenomenon is the so-called tunnelling effect, where electrons can be activated by thermal vibration and electron transition.
(3) Field emission theory: when there is a strong internal electric field among conductive fillers, an electric field emission current can be generated as electrons pass through the electronic barrier formed by the thin cementitious layer [92].

**Figure 9:**
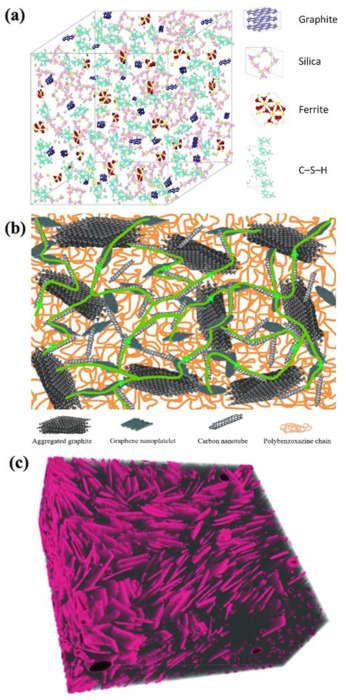
(**a**) conductive concrete mechanism (reprinted (adapted) with permission from [87] ©2021 with permission from Elsevier); (**b**) thermal conduction path (reprinted (adapted) with permission from [93] ©2020 with permission from Elsevier); (**c**) graphite flakes (pink) dispersed in matrix (gray), 3D reconstructed image of specimen (reprinted (adapted) from [3] which is an open access article and permits unrestricted use).

Sun et al. [87] analyzed the typical microstructures of ECCC samples with steel slag (SS), GP, and granulated blast-furnace slag (GGBS) fillers. A schematic diagram of the conductive concrete mechanism is given in Figure 9a, where conductive fillers (GP and SS) are evenly distributed and the C–S–H gel is well-filled in the aggregate framework. C–S–H gel plays an important role in improving both the mechanical and electrical performance of materials as it fills up the micropores of the mixture and tightens its bonds. SS containing ferrite can also improve ECCC conductivity. The optimized dispersion of these conductive components through the cementitious matrix can form privileged free conductive pathways. 

Witpathomwong et al. [93] reported on Polybenzoxazine (PBA) composites filled with three types of carbon filler for enhanced thermal conductivity: graphite, graphene, and CNTs. The dispersion of the fillers is blocked by the matrix, thus forming a barrier. When the amount of the conductive element increases to a critical value, the conductive networks expand to a certain range to form conductive paths (Figure 9b). Higher conductive pathways facilitate electron mobility, thus decreasing the resistivity of conductive concrete. These types of fillers can easily overlap and interlace with adjacent fillers, which can also create electrical conductive pathways [93]. Overlapped composite fillers play a critical role in the conductivity of composites.

Ioanna et al. [3] used μCT-scan technology to assess the dispersion of a 30 wt% graphite dose in a cement matrix. Figure 9c shows a 3D reconstructed image of the graphite-cement paste sample, where graphite flakes are well dispersed within the matrix and located near each other. Electric current can travel both through the conductive additive via “electronic conduction” and through the available free water via “electrolytic conduction”.

Many studies have shown that functional fillers can effectively enhance the electrical properties of cementitious composites. The ECCC is an interesting type of percolation system; its transport characteristics have attracted a great deal of research attention. The complex mechanisms of conduction suggest that the key to electrical conductivity is the formation of conductive pathways. The conductive pathways of cementitious composites filled with conductive fillers can be divided into three possible categories.

(1) Through the cement-based matrix: the electrical transport behavior of the cement-based composites is mainly affected by cement matrix system when the conductive component is lower than the percolation threshold value [94]. Electrical resistance is closely related to water consumption. Han et al. [95] found that electrical resistance decreases as water content increases, thus enhancing ionic conduction and ultimately improving electrical conductivity. Frattini et al. [64] reported that hardened cement paste with a relatively low added graphite content behaves as an insulator; the order of magnitude of its conductivity is approximately 10^−5^ S/m.
(2) Through composite conductive pathway: the composite conductive pathway is composed of a conductive component and cement matrix, so there is a synergic effect between the cementitious matrix and conductive fillers. The filler–matrix interface and C-S-H gel surface may be conductive insofar as improving the charge transfer mechanism [88]. Once graphite content reaches a certain level, the conductivity of GC composite pastes is of the order of magnitude from 10^−5^ to 1 S/m [64].
(3) Through the conductive network: once the conductive components in a cementitious composite form a conductive network, conductive fillers dominate electrical transport in the material. A higher conductive component content forms more continuous conductive pathways. A certain level of graphite content can bring the magnitude order of conductivity in GC composite pastes to between 1 and 10 S/m [64]. However, the conductive filler content should be controlled within a certain range to prevent the degradation of other concrete mixture properties.

### 8.2. Resistivity Testing Methods

Electrical resistivity (ρ) or conductivity (σ) are primary indicators of the electrical properties of materials. Electrical resistivity data must be accurately and precisely determined to characterize cementitious composites [86]. Currently, there is no standard or specification for the resistivity testing of cement-based composites. The selection and arrangement of electrodes significantly affects the conductive properties observed experimentally [45], and various methods produce variations in resistivity measurements [96]. Resistance-measuring methodology such as the specimen size, electrode material, and electrode embedded form affect the conductivity data of cementitious composites [86]. The resistivity test methods of the cement-based composites are summarized in Table 4.

**Figure 10:**
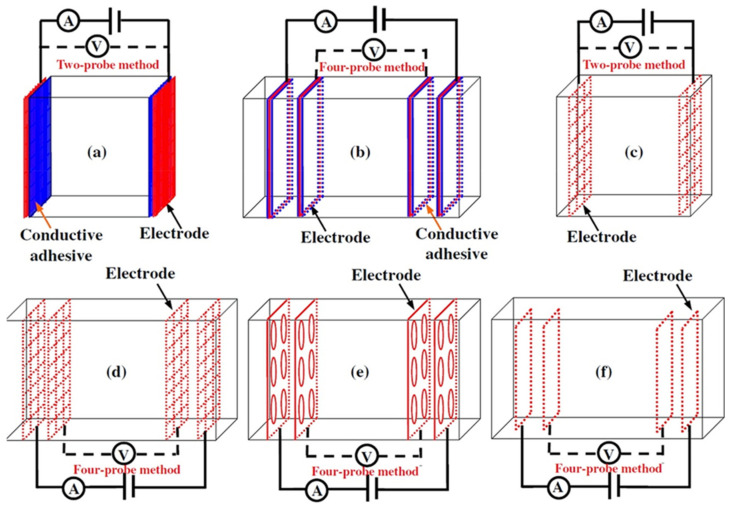
Schematic diagram of electrode configurations: (**a**) two-probe method, and (**b**) four-probe method with the attached electrode; (**c**) two-probe method, and (**d**) four-probe method with the copper mesh embedded electrode; (**e**), (**f**) the em-bedded electrode four-probe method with enlarged copper mesh opening (reprinted with permission from [8] ©2019 with permission from Elsevier).

(1) Specimen size: when small-size specimens are used, the discreteness and error of test values increase due to the inhomogeneity of the materials. Large specimen sizes are recommended for resistivity tests to improve uniformity and ensure the veracity of test data [97].
(2) Specimen treatment: fresh samples can be cured in molds for 24 h, then demolded and cured for 28 days (20 °C and 95% RH). After curing, such samples are usually processed in an oven for treatment to eliminate any polarization effect during resistivity measurements [98,99] and to minimize the influence of moisture and pore solution on the resulting volume resistivity data [100]. However, there is no universal standard for sample treatments. Samples may also be placed in an oven at 60 °C for three days followed by 95 °C for another three days [98], into an 105 °C oven for 24 h [100], or held overnight at 80 °C to eliminate free water [101].
(3) Test method: resistivity test methods include the two-probe method and four-probe method [8] (Figure 10). The 4-probe method has generally shown higher accuracy, as the 2-probe method may introduce contact resistance that results in error. The 2-probe method is more commonly used due to its relative convenience. However, the 4-probe method is recommended for the sake of accuracy [86].
(4) Test power supply: the supply voltage used to measure the resistivity of cementitious composites must fall within the resistivity stable region. Alternating current (AC) is recommended to measure the electrical resistance of the samples, as this can resolve the technical difficulties and problems (e.g., polarization effects) associated with direct current (DC) measurements [8,101].
(5) Test instrument: a high precision desktop digital multimeter is typically used in resistivity tests to reduce the influence of the test instrument on the resulting data.

**Table 4:** Electrical resistivity measurement methods.

Matrix	w/c	Specimen Size	Test Method	Test Power Supply	Test Instrument	Equation	Ref.
Paste	0.45	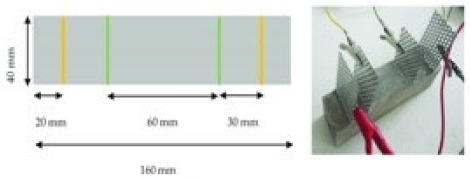 40 mm × 40 mm × 160 mm	4-probe method	10 V (DC)	-	-	[3]
Concrete	0.44	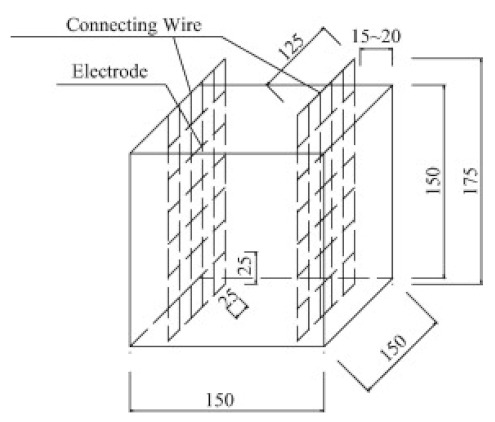 150 mm × 150 mm × 150 mm	2-probe method	50 Hz (AC)	Digital multimeter	-	[45]
Mortar		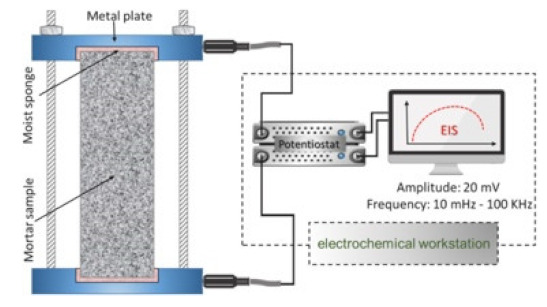 40 mm × 40 mm × 160 mm	Uniaxial two-point electrode method	20 mV 10 mKz-100 kHz (AC)	EIS tests (VMP3)	ρ = RS/L	[68]
Concrete	0.28	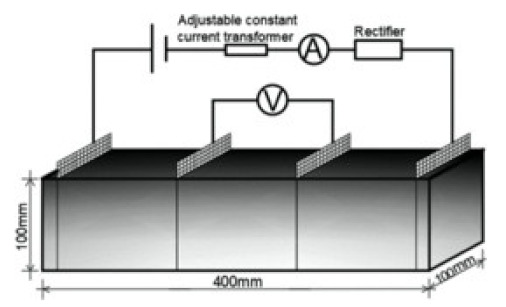 100 mm × 100 mm × 400 mm	4-probe method	-	Digital multimeter	ρ = 100 × UA/IL	[87]
Paste	0.4	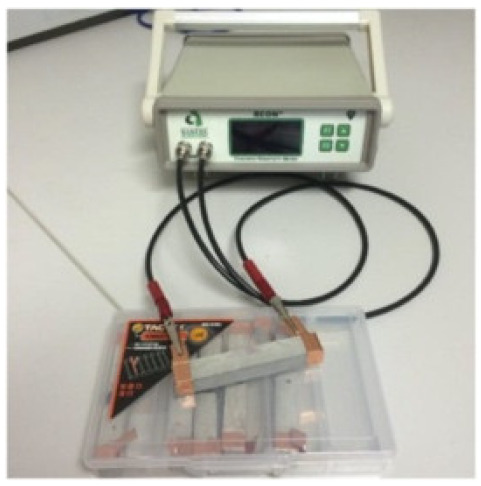 110 mm × 15 mm × 15 mm	2-probe method	1000 Hz(AC)	Resistivity meter	ρ = RAcosθ/L	[98]
Paste	0.5	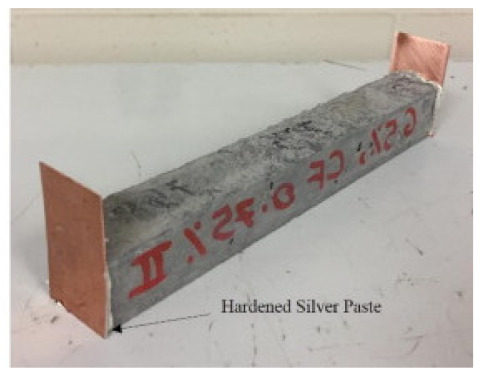 203.2 mm × 25.4 mm × 25.4 mm	2-probe method	DC	Digital multimeter and DC Hipot Tester	ρ = RS/L	[100]
Paste	0.45	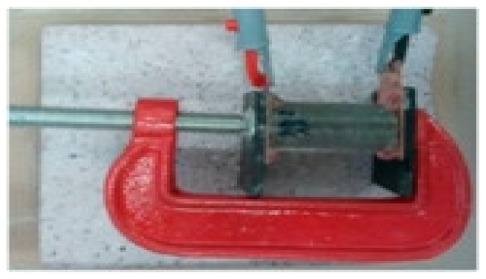 20 mm × 20 mm × 75 mm	2-probe method	300 mV 200 KHz (AC)	-	-	[101]
Concrete	0.43	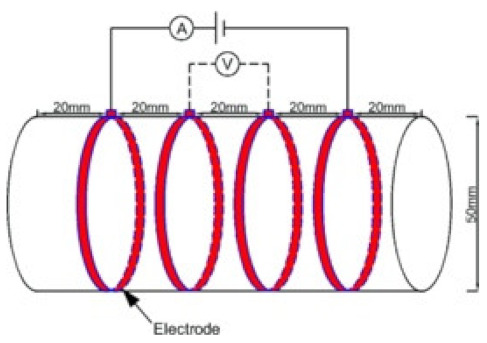 50 mm × 100 mm	4-probe method	DC	Digital multimeter	ρ = 2*πα*V/I	[102]
Paste	0.4	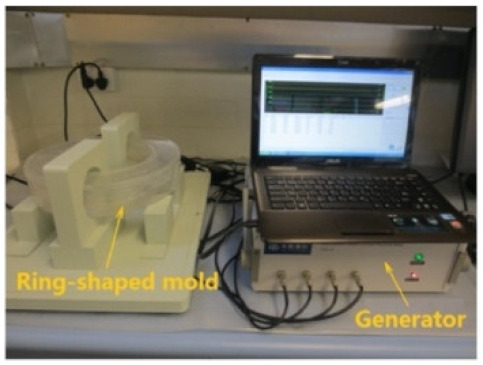 770 mm × 55 mm × 42 mm	Non-contact electrical resistivity test	-	Cement and Concrete Resistivity-III	-	[103]
Mortar	0.35	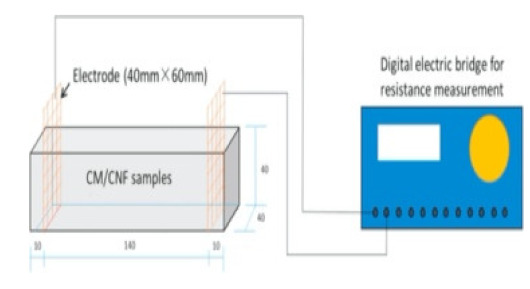 40 mm × 40 mm × 160 mm	2-probe method	AC	Digital multimeter	ρ = RS/L	[104]
Paste	0.35	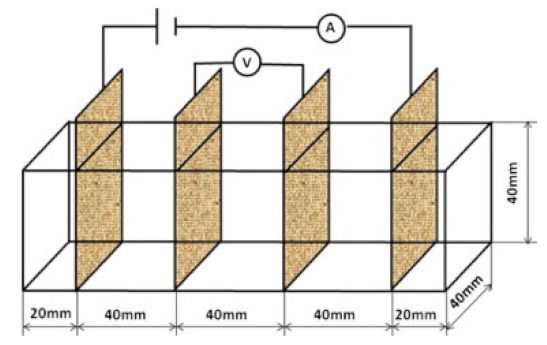 40 mm × 40 mm × 160 mm	4-probe method	DC	Digital multimeter	-	[105]

## 9. Methods to Improve Graphite-Based ECCC Properties

Carbon-based materials are currently the most often-used addition agents in the manufacture of ECCCs due to their excellent electrical conductivity and working stability. These materials involve CF, CB, and GP at the nanoscale. Despite their noteworthy potential as discussed above, they have technical limitations, yet restrict the practical utilization of carbon-based materials in cement composites.

ECCCs containing graphite-based fillers have negative effects in terms of physical microstructure, rheology, and mechanical behavior [87]. Considering the problems that emerge when utilizing graphite-based materials, many researchers have attempted different methods to improve their properties (e.g., fluidity, compatibility, mechanical properties, electrical conductivity). 

The compatibility between cement-based material and carbonic filler is poor, so effective dispersion technology and dispersing agents should be considered in establishing new ECCC designs. Surface modifications can be conducted with low-cost graphite materials to improve their dispersion and interfacial interactions in cementitious matrixes. Acid functionalization (a chemical modification) on the surface of graphite can introduce carboxyl and hydroxyl groups (-COOH and -OH), which creates uniform dispersion of graphite in the cement matrix and forms active sites for the initiation of cement hydration [106].

Graphite is comprised of conductive particles. Compared with fibrous material, however, it does not form conductive networks in a matrix as readily. To effectively increase conductivity, other types of conductive material (e.g., CNTs, CF, steel fiber) can be added to produce a multi-phase conductive matrix. These fiber-type fillers can further enhance the mechanical strength of the cementitious matrix to offset the low strength of graphite as well [45].

The ECCC design mixture should be optimized to balance mechanical and electrical properties. The use of slag as an admixture in carbon-based ECCCs can create workable tradeoffs among conductive properties, mechanical performance, cost-effectiveness, and environmental-friendliness [87]. 

## 10. Concluding Remarks and Future Research Directions 

Researchers have expressed interest in ECCCs for many years. The relatively low costs of graphite materials have made them attractive as potential ECCC additives for a variety of industrial purposes. This paper reviewed theoretical and experimental results relevant to graphite-based materials in the preparation of ECCCs. The main conclusions can be summarized as follows.

(1) The dispersion of graphite in the cement matrix is a notable technical limitation. The surfaces of graphite are hydrophobic and atomically smooth, thus encouraging mutual bonding to each other (i.e., agglomeration) in aqueous solutions (e.g., fresh cement mixtures).
(2) The properties of a fully fabricated ECCC are dependent on the quality of the filler dispersion. The size and dispersion of a given filler are more important than its conductivity. This dispersion may require further treatments such as surfactant addition to improve the final properties, or graphite may need further functionalization to achieve the desired properties.
(3) The ECCC is a heterogeneous material which has poor workability that restricts its wider application in engineering practice. A reduction in fluidity due to the inter-particle friction with cement particles, as well as the low hydrophilicity of graphite, cause a large amount of water to be entrapped in agglomerated graphite particles. The mixture design, water content, addition of any water reducing agents, graphite content, and fineness should be adjusted to ensure sufficient flowability without sacrificing functionality.
(4) Graphite does not directly participate in cement hydration; rather, graphite particles act as inert conductive fillers. Graphite has a large specific surface area which can provide nucleation sites for hydration product precipitation. A large amount of hydration products is generated near the graphite sheets, which may improve the compatibility of graphite as a cement composite additive.
(5) The key parameter of an ECCC is its electrical conductivity. Electrical conductivity increases as graphite addition increases, but compressive strength decreases simultaneously. The cementitious matrix–graphite particle interface has a significant effect on compressive strength. The graphite content supplied to a cementitious system must be properly adjusted to minimize any adverse effects on the mechanical properties of the material.
(6) The addition of graphite in the matrix increases its porosity. Graphene-based materials as fillers not only create a physical barrier, but also form tortuous network paths that ultimately reduce the permeability of the composite.
(7) The high surface area of fillers allows them to efficiently control the propagation of microcracks in cementitious composite materials. The layered structure of graphite further allows it to entrap ions which can protect the matrix.
(8) The long-term performance (e.g., freeze–thaw resistance, shrinkage, sulfate resistance, steel corrosion resistance) of graphite-based cementitious composites has not yet been reported. To effectively utilize graphite in future engineering practice, in-depth research on other properties of ECCCs with graphite are yet needed.
(9) The ECCC is a percolation system with complex conduction mechanisms that have attracted a great deal of research attention. The electrically conductive mechanisms of cementitious composites need further research in regard to their transport and electrical conductivity properties.
(10) Currently, there is no strict standard or specification for ECCC conductivity testing. Electrical resistivity is the primary index of ECCCs, which determines its performance and application value. A standardized test method for ECCC electrical resistivity is of great significance in terms of the material’s potential application in engineering practice.

Graphite-based cementitious composites have shown excellent performance in previous studies, but challenges persist. The successful use of graphite in ECCCs requires adequate dispersion in the aqueous fresh mixture to ensure that an electrically conductive network forms within the cementitious structure, sufficient workability for practical engineering, and adequate bonding of cement hydration products for effective stress transfer across the interfaces. The physical or chemical modification of graphite-based materials can enhance the overall performance of the cementitious matrix. It is necessary to further research graphite-based material modification technologies to support the usage of conductive carbon fillers in the construction industry, and in turn to extend the possible applications of ECCCs.

## Figures and Tables

**Table 1:** General properties of carbon materials [8,9].

Carbon Material	State	Bulk Density (g/cm^3^)	Specific Surface Area (m^2^/g)	Conductivity (S/cm)	Dispersion	Cost
CNFs	Fiber	0.06–2.1	13–200	10–10^4^	Aggregates easily	High
CFs	Fiber	1.5–2.0	10–50	10^−1^–10^3^	Aggregates easily	Medium
Graphene	Powder	1–2.5	120–575	10^3^	Relatively easier dispersion	High
GP	Powder	1.9–2.3	10–35	10^4^	Relatively easier dispersion	Low
CB	Powder	0.4–2.0	20–250	10	Aggregates easily	Low

## Data Availability

Data sharing not applicable. No new data were created or analyzed in this article. Data sharing is not applicable to this article.
